# Bioterrorism-Related Anthrax: International Response by the Centers for Disease Control and Prevention

**DOI:** 10.3201/eid0810.020345

**Published:** 2002-10

**Authors:** Christina S. Polyak, Jonathan T. Macy, Margarita Irizarry De La Cruz, James E. Lai, Jay F. McAuliffe, Tanja Popovic, Segaran P. Pillai, Eric D. Mintz, the Emergency Operations Center International Team

**Affiliations:** * Centers for Disease Control and Prevention, Atlanta, Georgia, USA; † Department of Health, Bureau of Laboratories, Miami, Florida, USA

**Keywords:** anthrax, bioterrorism, international, *Bacillus anthracis*

## Abstract

After reports of the intentional release of *Bacillus anthracis* in the United States, epidemiologists, laboratorians, and clinicians around the world were called upon to respond to widespread political and public concerns. To respond to inquiries from other countries regarding anthrax and bioterrorism, the Centers for Disease Control and Prevention established an international team in its Emergency Operations Center. From October 12, 2001, to January 2, 2002, this team received 130 requests from 70 countries and 2 territories. Requests originated from ministries of health**,** international organizations, and physicians and included subjects ranging from laboratory procedures and clinical evaluations to assessments of environmental and occupational health risks. The information and technical support provided by the international team helped allay fears, prevent unnecessary antibiotic treatment, and enhance laboratory-based surveillance for bioterrorism events worldwide.

Immediately following reports of the intentional release of *Bacillus anthracis* in the United States in October 2001, public health professionals around the world were called upon to respond to widespread political and public concerns. Specific threats, hoaxes, and incidents in other countries directly affected U.S. institutions and citizens, as well as expatriate U.S. government employees, businessmen, journalists, and travelers. The Centers for Disease Control and Prevention (CDC) established an international team in its Emergency Operations Center to respond to inquiries from other countries regarding anthrax and bioterrorism.

## Methods

The international team included physicians, microbiologists, epidemiologists, and other public health officials with expertise in international affairs and infectious diseases. Team members were fluent in several languages. From October 12, 2001, to January 2, 2002, the team provided rapid feedback and support in response to requests for assistance on bioterrorism-related topics. The team was available for consultation by telephone and e-mail, 24 hours a day, 7 days a week. The team electronically disseminated documents on anthrax and bioterrorism preparedness and collaborated with the World Health Organization (WHO) and its regional offices to facilitate exchanging relevant information.

Requests for assistance were classified into four general categories: laboratory-related issues, general bioterrorism information, environmental and occupational concerns, and bioterrorism preparedness. Depending on the nature of the request, the team sought assistance from other CDC experts.

The level of support provided to various countries for specific requests was divided into two categories: high or medium. High, or technical, support included one or more of the following: testing clinical and environmental or nonclinical specimens and isolates, arranging for specimens and isolates to be tested at a reference laboratory, coordinating with CDC staff in**-**country to provide on-site consultation and assistance, and providing reagents for performing microbiological tests. Medium, or informational support, included telephone or e-mail consultation regarding bioterrorism, laboratory methods, and preparedness.

## Results

**Figure 1:**
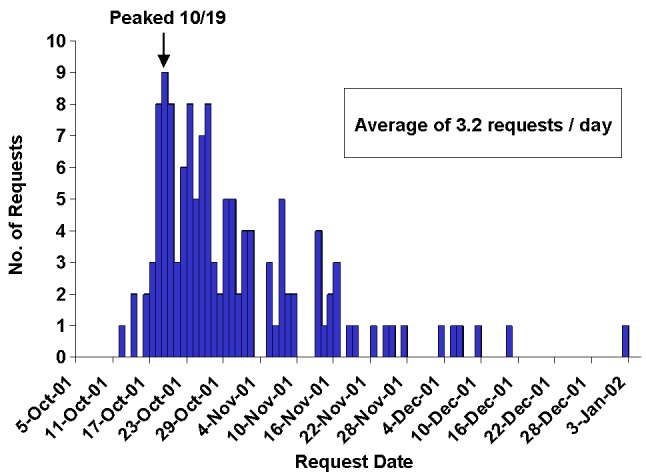
Requests for assistance to international team of the Emergency Operations Center, October 12, 2001–January 2, 2002 (n=130).

The international team received 130 requests for assistance from 70 countries and 2 territories during the period October 12, 2001, to January 2, 2002. An average of 3.2 requests per day (with a peak of 9 requests on October 19) were received by e-mail (55.4%) and telephone (44.6%) (Figure 1). Forms of support provided to other countries included consultation regarding laboratory methods for isolation and identification of *B. anthracis*, clinical and epidemiologic support, and policy and preparedness. Of the 130 requests, 54 (41.5%) were laboratory related; 51 (39.2%) were general requests for bioterrorism information; 14 (10.8%) were for environmental or occupational health guidelines; and 11 (8.5%) concerned developing bioterrorism-preparedness plans. Ninety-three (71.5%) of the requests were from persons or agencies affiliated with Ministries of Health; 15 (11.5%) were from other public health or medical professionals; 13 (10.0%) were from private citizens; and 9 (6.9%) were from international organizations such as WHO and the Pan American Health Organization. Requests were not evenly distributed by region. Europe and Latin America/the Caribbean each accounted for 25.4% of the total requests, followed by Asia (22%) and Africa (15%).

**Figure 2:**
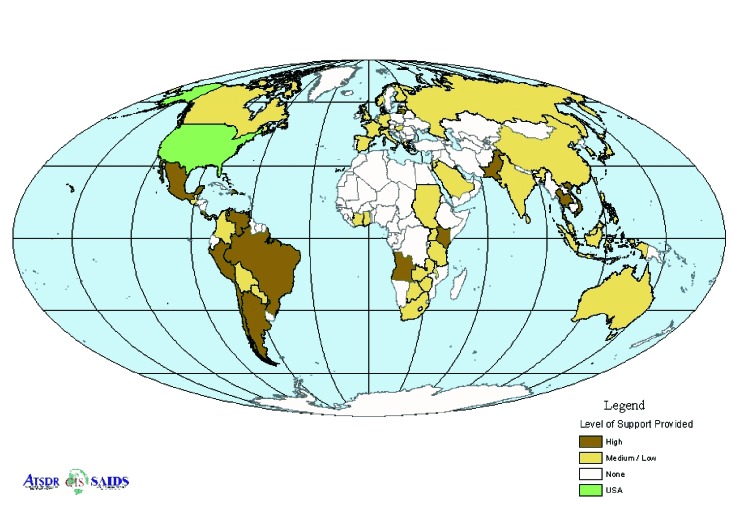
International response: level of support provided.

**Figure 3:**
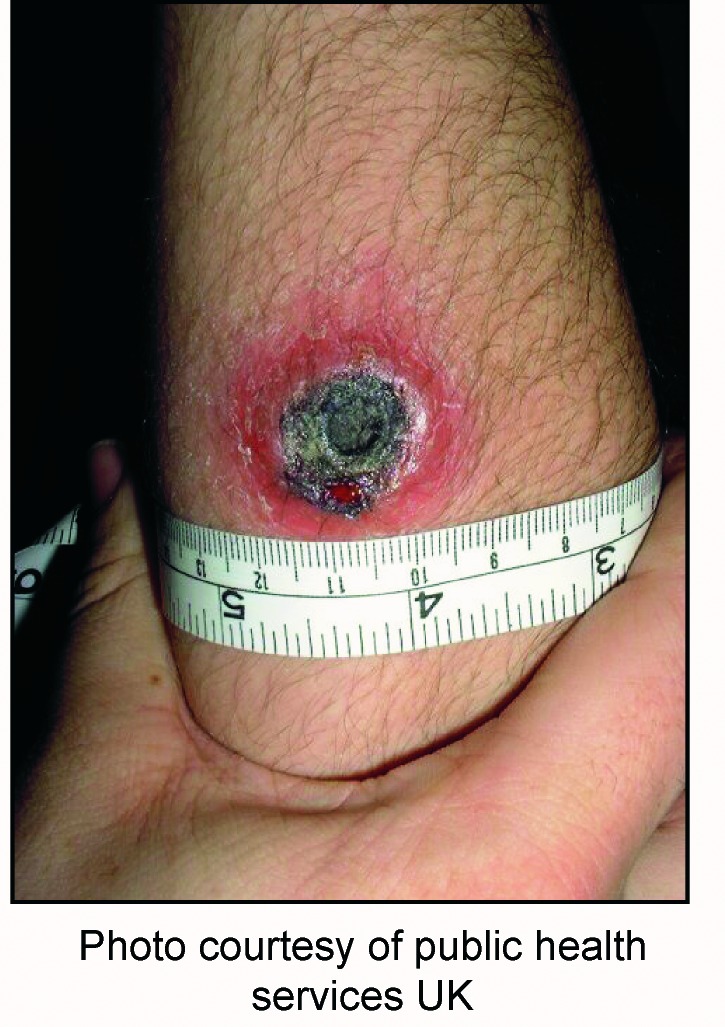
Suspected cutaneous anthrax lesion from a patient in the United Kingdom. Photos like this, transmitted by e-mail, enabled clinical experts to review images of suspected cases worldwide and provide rapid assistance.

Of the 70 countries and 2 territories, 55 (76.4%) received informational support or telephone or e-mail consultation regarding bioterrorism events or preparedness (Figure 2). The remaining 17 (23.6%) received a high or technical level of support, including testing specimens at a Laboratory Response Network member reference laboratory (n=12). Digital images of suspected *B. anthracis* isolates and cases were submitted by e-mail, which enabled laboratory and clinical experts to review images of suspected cases worldwide and provide rapid guidance (Figure 3). All images of isolates and cases sent were subsequently found to be negative.

Four isolates from outside the United States were confirmed as *B. anthracis*. Three of these isolates were cultured from mail sent by the U.S. Department of State to U.S. embassies in Lima, Peru (two), and Vienna, Austria (one). These three isolates were indistinguishable from all other U.S. outbreak isolates by molecular subtyping by multilocus variable number of tandem repeats typing. An additional isolate, recovered by the Chilean National Institute of Public Health from a letter to a private physician in Chile, was a different subtype from those in the U.S. outbreak. The source of this isolate is being investigated.

The team’s active role concentrated on information dissemination and collaboration with WHO. Documents on anthrax and bioterrorism were prepared and disseminated electronically to all CDC international assignees (41 countries), to epidemiologists and laboratorians affiliated with the Training Programs in Epidemiology and Public Health Interventions Network (TEPHINET, 33 countries), and to the WHO Global Salmonella Surveillance List Serve (Global Salm-Surv, 106 countries). Electronic dissemination allowed rapid distribution and availability of contact information for the team. In addition, the Morbidity and Mortality Weekly Report (MMWR) published a short statement about the team’s activities that described assistance to other countries and directed questions regarding bioterrorism-related issues outside the United States to the team (1). Collaboration with WHO regional offices included the development and support of a training course on the management of suspected exposures to anthrax spores (2). Representatives from 14 countries attended the course, which was conducted in Bangkok in December 2001 and was sponsored by the CDC International Emerging Infections Program in Thailand and the WHO headquarters Southeast Asia Regional Office. In addition, the international team worked with WHO to develop a database of laboratories capable of serving as anthrax reference laboratories in various countries throughout the world.

## Discussion

Any suspected bioterrorism event has immediate global implications, no matter who the intended target or where the event occurs. This global impact is particularly true for communicable diseases such as smallpox. However, because of international trade, travel, and social connectedness, the same principle applies to less easily transmitted communicable diseases such as anthrax. More than 4,000 threat letters were tested in public health laboratories in Europe in the month after the first report of intentionally contaminated mail in the United States, and all surveyed national public health institutions took extraordinary measures to improve bioterrorism preparedness (3).

In countries throughout the world, threat letters caused a shift in resources from traditional public health concerns to national security concerns. This shift represents a particular challenge for developing countries with chronically scarce resources for public health. Therefore, additional resources, particularly for the health sector of developing countries, are needed to address future threats. In many countries, strengthening the public health surveillance and response capacity for naturally occurring emerging infectious diseases is the most efficient means to provide a critical early warning system for intentionally released biologic agents and a defense against their further spread.

Public health agencies need to be able to exchange information rapidly across international borders to keep pace with events and make critical medical and public health decisions. Public health agencies must also keep pace with worldwide media coverage to minimize the potential for misguided public reaction. In the United States and other countries, many persons who were exposed to suspected anthrax-containing materials were told to not start or to discontinue antibiotic chemoprophylaxis after anthrax exposure was ruled out by testing at public health reference laboratories and by further epidemiologic investigations (4). Information and technical support provided by the international team helped allay fears, prevent unnecessary antibiotic treatment, and enhance laboratory-based surveillance for bioterrorism events worldwide.

The operations of the international team were not without difficulty. Responding rapidly in different languages to countries in different time zones proved to be a challenge. In addition, the team was not always able to provide rapid technical assistance because of the need for review and clearance of documents containing new scientific information. Despite strict adherence to regulations governing the transport of infectious agents, shipment of suspected isolates of *B. anthracis* from laboratories in one country to reference laboratories in the United States was complicated by hesitance from shipping companies, air carriers, and national authorities. In some cases, the laboratory investigation of suspected exposures was delayed for several days while consent was sought from higher authorities and willing shipping companies and air carriers were identified.

The largest percentage of requests received by the international team were from persons or agencies affiliated with ministries of health, reflecting concern about bioterrorism issues at the national government level. On request, the team also provided specific information about the events occurring in the United States, often through referrals to publications and other materials regularly posted on the CDC website. Information provided to field epidemiology training programs through TEPHINET addressed some of these issues proactively and reduced the overall number of requests (5). Given the essential role of the public health laboratory in bioterrorism preparedness and response (6), information provided proactively to laboratories through the WHO Global Salm-Surv listserv may also have reduced the number of requests.

Rapid, reliable access to the Internet is an extremely useful tool for connecting public health agencies and laboratories and should be universally promoted. Digital cameras are an economical means of capturing clinical and laboratory images for Internet transmission and can greatly enhance communication about suspected cases or specific etiologic agents of infectious diseases. Nonclassified commercial laboratory reagents and protocols for isolating and identifying *B. anthracis* and other bioterrorism agents should be widely available to national public health reference laboratories. Through its collaborating centers, WHO has already begun to establish a worldwide network of reference laboratories capable of isolating, identifying, and confirming bioterrorism agents; WHO will continue to play a critical role in global coordination of outbreak surveillance and response. In addition, during the World Health Assembly of May 2002, the 191 member states agreed to a resolution recognizing that a deliberate release of biological agents could have serious public health implications and jeopardize public health achievements of the past decades (7).

In the long term, strengthening the capabilities of national public health agencies and laboratories to recognize and respond to potential bioterrorist events and agents will also build capacity for recognition and response to naturally occurring outbreaks. Ensuring connectivity between these national public health agencies and reference laboratories worldwide is critical to improving global preparedness for emerging infectious diseases, whether or not they result from the deliberate release of a bioterrorism agent.

## References

[1] Centers for Disease Control and Prevention. Update: Investigation of bioterrorism-related anthrax, 2001. MMWR Morb Mortal Wkly Rep. 2001;50:1008–10. 11724158

[2] Simmerman JM, Kumari S, Bhatia R, Dejsirilert S, Sangsuk L, Sawanpanyalert P, Strengthening public health response to anthrax in Southeast Asia. Proceedings of the 10th International Conference on Infectious Diseases 2002, March 11–14, 2002. Singapore.

[3] Coignard B. Bioterrorism preparedness and response in European public health institutes. Eurosurveillance. 2001;6:159–66. 11891386 10.2807/esm.06.11.00383-en

[4] Centers for Disease Control and Prevention. Notice to readers: interim guidelines for investigation of and response to *Bacillus anthracis* exposures. MMWR Morb Mortal Wkly Rep. 2001;50:987–90. 11724154

[5] Sandhu HS, Thomas C, Nsubuga P, White M, Mendlein J, Maes E, Assessment of bioterrorism response capacity of applied epidemiology and training programs—2001. Proceedings of International Conference on Emerging Infectious Diseases 2002, March 24, 2002. Atlanta, Georgia.

[6] Khan A, Morse S, Lillibridge S. Public health preparedness for biological terrorism in the USA. Lancet. 2000;356:1179–82. 10.1016/S0140-6736(00)02769-0 11030310

[7] World Health Organization. Global public health response to natural occurrence, accidental release or deliberate use of biological and chemical agents or radionuclear material that affect health. Geneva: 55th World Health Assembly; May 18, 2002.

